# Author Correction: Two-magnon scattering in the 5*d* all-in-all-out pyrochlore magnet Cd_2_Os_2_O_7_

**DOI:** 10.1038/s41467-017-01513-4

**Published:** 2017-11-17

**Authors:** Thi Minh Hien Nguyen, Luke J. Sandilands, C. H. Sohn, C. H. Kim, Aleksander L. Wysocki, In-Sang Yang, S. J. Moon, Jae-Hyeon Ko, J. Yamaura, Z. Hiroi, Tae Won Noh

**Affiliations:** 1 Center for Correlated Electron Systems, Institute for Basic Science (IBS), Seoul, 151-742 Republic of Korea; 2 Department of Physics and Astronomy, Seoul National University (SNU), Seoul, 151-742 Republic of Korea; 3 Ames Laboratory, U.S. Department of Energy, Ames, IA 50011 USA; 4 Department of Physics and Division of Nano-Sciences, Ewha Womans University, Seoul, 120-750 Republic of Korea; 5 Department of Physics, Hanyang University, Seoul, 133-791 Republic of Korea; 6 Department of Physics, Hallym University, Chuncheon, Gangwondo 24252 Republic of Korea; 7 Materials Research Center for Element Strategy, Tokyo Institute of Technology, Kanagawa, 226-8503 Japan; 8 Institute for Solid State Physics, University of Tokyo, Kashiwa, 277-8581 Japan; 9 Present Address: Measurement Science and Standards, National Research Council of Canada, Ottawa, Ontario Canada K1A 0R6

*Nature Communications* **8**:251 doi:10.1038/s41467-017-00228-w; Article published online 15 August 2017

This Article contains typographical errors in Equation (2), where two division operators were omitted. The correct version of Equation (2) appears below

$$I\left( \omega \right) = I_0\frac{{\left[ {1 + \left( {\omega - \omega _0} \right)/q\Gamma } \right]^2}}{{1 + \left[ {\left( {\omega - \omega _0} \right)/\Gamma } \right]^2}}.$$

