# Supplementary material for: Two-magnon scattering in the 5d all-in-all-out pyrochlore magnet Cd2Os2O7
Source: Nat Commun. 2017 Aug 15;8:251. doi: 10.1038/s41467-017-00228-w (PMC5557926; doi:10.1038/s41467-017-00228-w)
Supplement: Supplementary file 1 — Supplementary Information [file 41467_2017_228_MOESM1_ESM.pdf]

File name: Supplementary Information
Description: Supplementary Figures, Supplementary Notes, Supplementary Table and Supplementary References

File name: Peer Review File
Description:

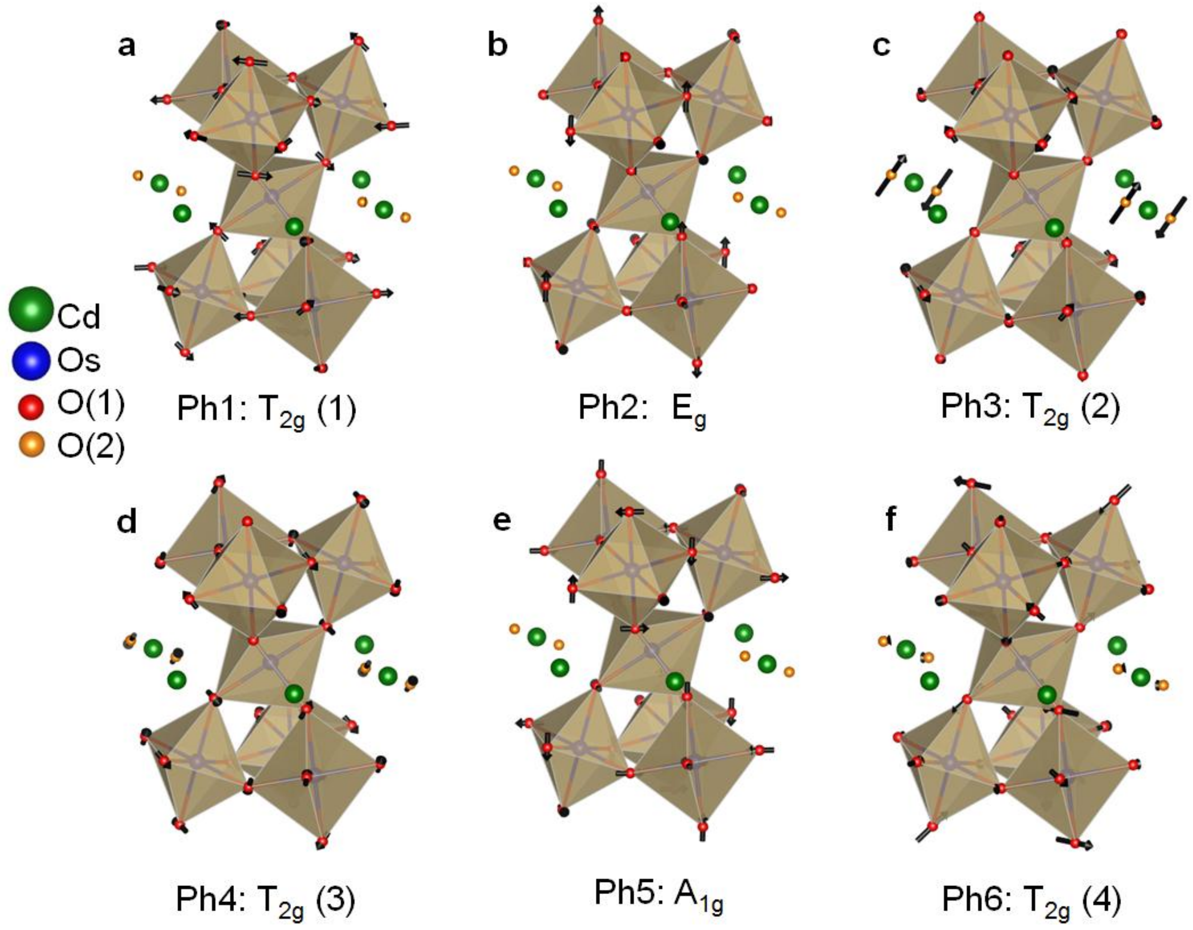

Supplementary Figure 1. **The ionic displacement of 6 phonon modes in $Cd_2Os_2O_7$**. The Cd ions are in green. Trigonally compressed cages of oxygens O(1) (red) surround each Os, and the oxygens O(2) (yellow) are bonded to Cd.

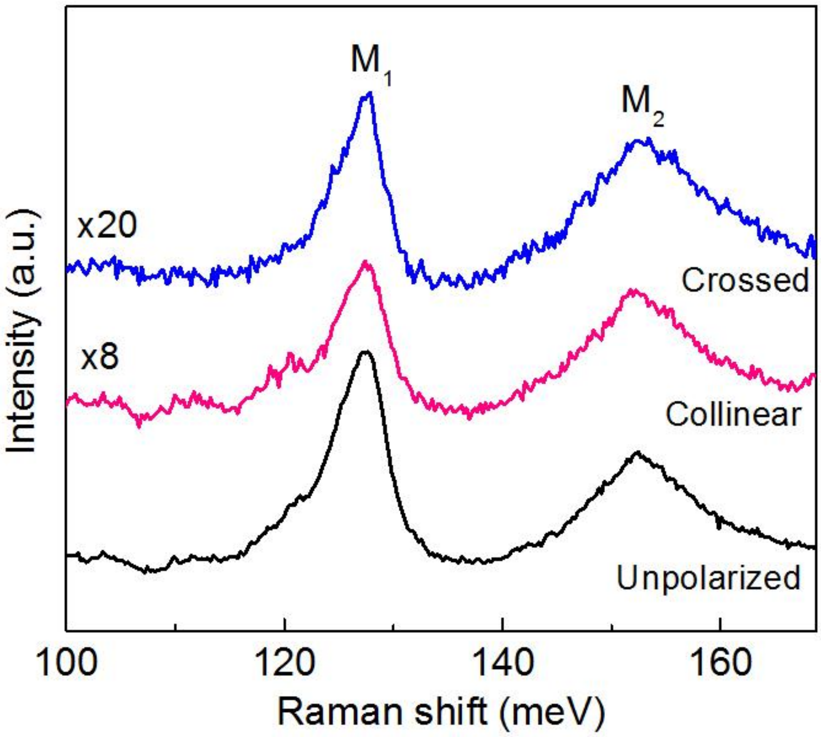

Supplementary Figure 2. **Polarized Raman spectra of a pyrochlore $Cd_2Os_2O_7$ single crystal**. The measurements are obtained at 15K in three input-output polarization geometries: crossed, collinear and unpolarized.

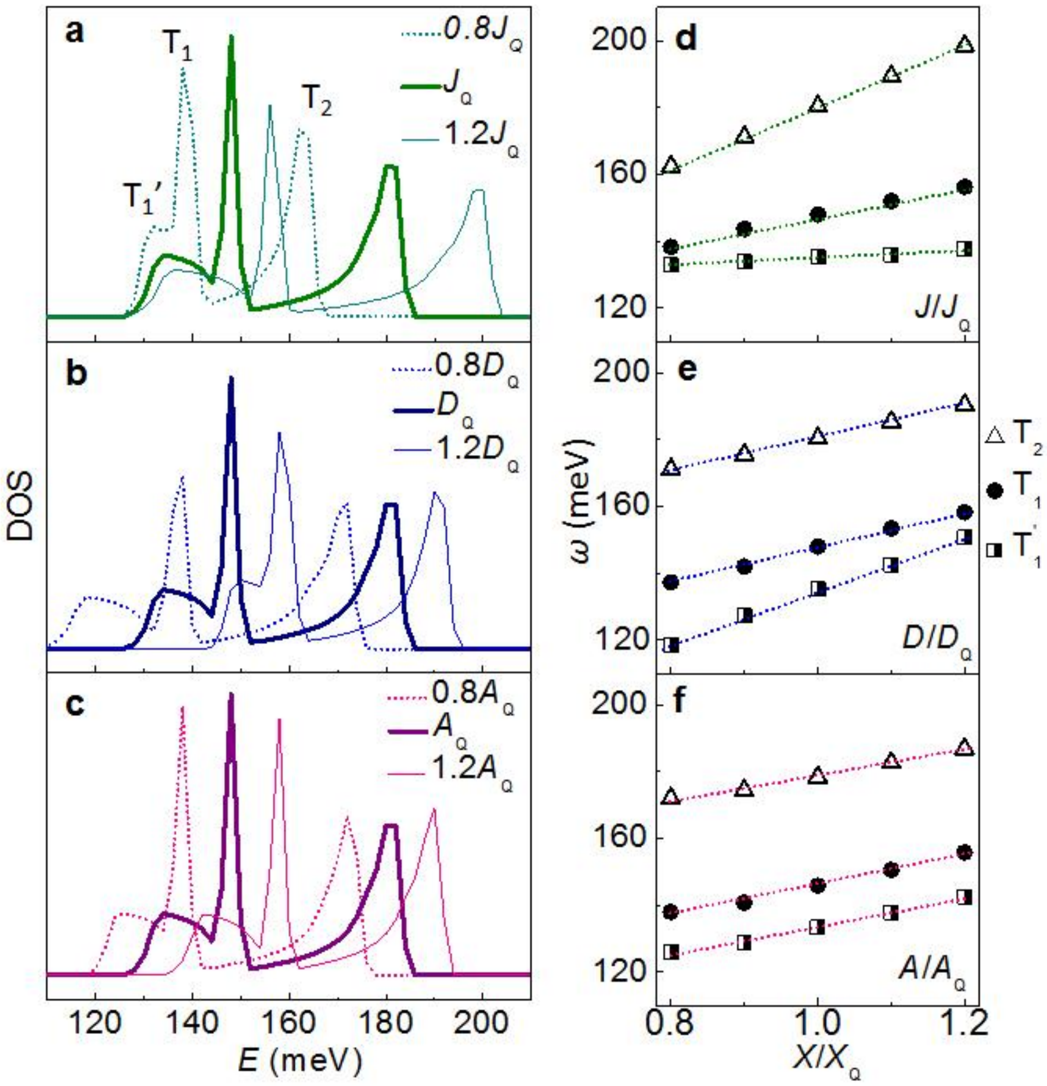

Supplementary Figure 3. **LSWT calculations of the effect of *J*, *D* and *A* on magnetic excitation.** (**a - c**) The magnon density of states (DOS) calculations with different values of Heisenberg exchange integral (**a**), Dzyaloshinskii-Moriya (DM) interaction (**b**), and single ion anisotropy (SIA) (**c**). (**d-f**) is the relation between *J* (**d**), *D* (**e**), and *A* (**f**) and energy of the two-magnon T_1', T_1 and T_2. The subscript Q denotes the qualities calculated by linear spin wave theory with J_Q = 6.4 meV, D_Q = 1.7 meV and A_Q = -6.8 meV. These values were reported from earlier many-body quantum mechanical calculations[1]. X denotes *J*, *D* and *A* in (d) (e) and (f), respectively.

Supplementary Note 1: First-principles calculations and phonon mode assignments

First-principles calculations are used to assign the observed six Raman-active phonon modes. The first-principles calculations were performed using the density functional theory + U method with the PBEsol form of exchange correlation functional as implemented in the Vienna Ab initio Simulation Package (VASP). We used frozen phonon method for calculating zone-center phonon. We choose U_{eff} = 1.5 eV to model the local Hubbard interaction in Os and the AIAO magnetic ordering is considered within the noncollinear density functional theory formalism.

Supplementary Table 1 shows the frequencies of six phonon modes from the calculations and from Raman spectra at 15 K. The experimental data show good agreement with the theoretical values. By analysing the main ionic motion associated with each mode, we can conclude that the Cd and Os ions, whose site symmetry is D_{3d}, do not participate in the Raman active modes. O(1), the oxygen atom at the apex of a OsO_6 octahedron with site symmetry C_{2v} results in one A_{1g}, one E_g and three T_{2g} Raman-active phonon modes [T_{2g}(1), T_{2g}(3) and T_{2g}(4)]. O(2), the oxygen bonded to Cd, gives one T_{2g} mode [T_{2g}(2)]. The associated eigenmodes are shown in Supplementary Fig. 1.

Supplementary Note 2: Effect of *J*, *D* and *A* on magnetic excitation

To look for the best agreement between the experimental spectra and the calculated magnon density of states (DOS), we study the effect of the exchange interaction (J), DM interaction (D) and SIA (A) on the magnon DOS. Supplementary Fig. 3 shows that decreasing J, D and A will decrease the energy of each two-magnon peak. However, the splitting between the three two-magnon peaks depends on J but is almost not affected by A. D only affects the splitting between T_1' and T_1, but does not have role in the splitting between T_1 and T_2.The exchange interaction J also affects the width of the magnon DOS, consistent with a change in the magnon dispersions, but leaves the onset in the DOS unchanged. Thus, the magnetic couplings are unambiguously fixed by the experimental data. A linear relation between the J, D and A values and the energy of T_1', T_1 and T_2 are observed in Supplementary Fig. 3d-f. Experimentally, the two-magnon peaks M_1', M_1 and M_2 are located at ~ 121 meV, 127 meV and ~152 meV, respectively. The splitting between M_1' and M_1 is ~6 meV and the splitting between M_1 and M_2 is ~ 25 meV. We first calculated the magnon DOS using LSWT with J_Q = 6.4meV, D_Q = 1.7 meV and A_Q = -6.8meV where the subscript Q denotes values from

many-body quantum chemical calculations[1]. The DOS shows three two-magnon peaks T_{1Q}', T_{1Q} and T_{2Q} at 135 meV, 148meV and 180meV, respectively. Compared with the experimental spectra, the splitting obtained using the quantum chemical results between T_1' and T_1 is larger than the splitting between M_1' and M_1 by ~7meV, and the splitting between T_1 and T_2 larger than the splitting between M_1 and M_2 by the same value (~7meV). As can be seen in Supplementary Fig. 3d-f, decreasing J will decrease both of the splitting between T_{1Q} and T_{2Q}, as well as the splitting between T_{1Q}' and T_{1Q}. Then, by changing the value of A, we can change the energy of three DOS peaks without affecting their splitting. We found that the data is well-described using J, D and A values of 5.1 meV, 1.7 meV, and -5.3 meV, respectively.

Supplementary Table 1

Phonon frequencies from first-principles calculations, Raman peak frequencies at 15K, and assignments of the modes.

	Modes	DFT (meV)	ω_R (80K) (meV)	Assignment
Ph1	$T_{2g}(1)$	26.2	28	O(1)-Os
Ph2	E_g	29.0	32	O(1)-Os
Ph3	$T_{2g}(2)$	41.9	43	O(2)-Cd
Ph4	$T_{2g}(3)$	54.1	57	O(1)-Os
Ph5	A_{1g}	57.4	60	O(1)-Os
Ph6	$T_{2g}(4)$	97.6	96	O(1)-Os

Supplementary References

1. Bogdanov, N. A., Maurice, R., Rousochatzakis, I., Brink, J. V. D.& Hozoi, L., Magnetic State of Pyrochlore $Cd_2Os_2O_7$ Emerging from Strong Competition of Ligand Distortions and Longer-Range Crystalline Anisotropy, *Phys. Rev. Lett*, **110**, 127206, (2013).
